# Statins Inhibit Angiotensin II/Smad Pathway and Related Vascular Fibrosis, by a TGF-β-Independent Process

**DOI:** 10.1371/journal.pone.0014145

**Published:** 2010-11-30

**Authors:** Raúl Rodrigues Díez, Raquel Rodrigues-Díez, Carolina Lavoz, Sandra Rayego-Mateos, Esther Civantos, Juan Rodríguez-Vita, Sergio Mezzano, Alberto Ortiz, Jesús Egido, Marta Ruiz-Ortega

**Affiliations:** 1 Cellular Biology in Renal Diseases Laboratory, Universidad Autónoma de Madrid, Madrid, Spain; 2 Division of Nephrology, School of Medicine, Universidad Austral, Valdivia, Chile; 3 Dialysis Unit, Fundación Jiménez Díaz, Madrid, Spain; 4 Renal Research Laboratory, Fundación Jiménez Díaz, Universidad Autónoma de Madrid, Madrid, Spain; Inserm, France

## Abstract

We have recently described that in an experimental model of atherosclerosis and in vascular smooth muscle cells (VSMCs) statins increased the activation of the Smad pathway by transforming growth factor-β (TGF-β), leading to an increase in TGF-β-dependent matrix accumulation and plaque stabilization. Angiotensin II (AngII) activates the Smad pathway and contributes to vascular fibrosis, although the *in vivo* contribution of TGF-β has not been completely elucidated. Our aim was to further investigate the mechanisms involved in AngII-induced Smad activation in the vasculature, and to clarify the beneficial effects of statins on AngII-induced vascular fibrosis. Infusion of AngII into rats for 3 days activates the Smad pathway and increases fibrotic-related factors, independently of TGF-β, in rat aorta. Treatment with atorvastatin or simvastatin inhibited AngII-induced Smad activation and related-fibrosis. In cultured rat VSMCs, direct AngII/Smad pathway activation was mediated by p38 MAPK and ROCK activation. Preincubation of VSMCs with statins inhibited AngII-induced Smad activation at all time points studied (from 20 minutes to 24 hours). All these data show that statins inhibited several AngII-activated intracellular signaling systems, including p38-MAPK and ROCK, which regulates the AngII/Smad pathway and related profibrotic factors and matrix proteins, independently of TGF-β responses. The inhibitory effect of statins on the AngII/Smad pathway could explain, at least in part, their beneficial effects on hypertension-induced vascular damage.

## Introduction

Hypertension causes structural changes in the arteries (vascular remodeling) that involve alterations in cell growth, vascular smooth muscle cell (VSMCs) hypertrophy and accumulation of extracellular matrix (ECM) [1], [2]. Among the factors involved in these processes, AngII has a key influence in the architecture and integrity of the vascular wall, by its role as a true cytokine that regulates cell growth, inflammation and fibrosis [1]–[3].

The molecular mechanisms involved in AngII signaling are complex, including activation of transcription factors, protein kinases and redox process [1]–[3]. The Smad pathway is the main signaling system of transforming growth factor-β (TGF-β), but growing evidence suggests that other factors can directly activate this intracellular pathway [4]. Several *in vitro* studies have shown that AngII activates Smad pathway independently of TGF-β in cultured VSMCs [5], [6] and other cell types [7]–[9]. Infusion of AngII into rats induced aortic Smad activation [5], [6], [10], but its relation with TGF-β and vascular fibrosis has not been completely elucidated. In VSMCs, AngII, after binding to AT_1_ receptors, increases the phosphorylation of regulated-Smad (Smad2 or Smad3) that binds to Smad4, then this complex is translocated into the nucleus where it acts as a transcription factor and upregulates Smad-dependent gene transcription, including fibrotic-related genes, such as connective tissue growth factor (CTGF) and collagens [5], [6].

The inhibitors of the 3-hydroxy-3-methylglutaryl coenzyme A (HMG-CoA) reductase (also called statins) exert beneficial effects in cardiovascular diseases. Besides their well-known effects in downregulation of circulating cholesterol, statins also exert pleiotropic effects at the cellular level, regulating intracellular signaling systems [11]. We have recently demonstrated that in cultured VSMCs, statins increased TGF-β-mediated Smad activation and upregulated TGF-β receptor type II expression, leading to an increase of TGF-β-mediated responses, including ECM upregulation [12]. There are few studies evaluating the *in vivo* effect of statins on Smad pathway. In experimental models of atherosclerosis in mice, atorvastatin activates Smad signaling and increased collagen deposition in the atheroma plaques [12], [13]. However, antifibrotic proterties of statins have been described, such as inhibition of AngII-induced vascular fibrosis [14].

Our aim was to investigate the mechanisms involved in AngII-induced Smad activation in the vasculature, and the effect of HMG-CoA reductase inhibitors in this pathway. The investigation of the molecular mechanisms involved in these processes could lead to a better understanding of cardiovascular pathology and to optimize therapeutic strategies.

## Methods

### Experimental studies

Systemic infusion of AngII (100 ng/kg per minute; subcutaneous osmotic minipumps; Alza Corp) was performed into male Wistar rats of 3 months of age for 3 days. Some animals were also daily treated with the HMG-CoA reductase inhibitors atorvastatin and simvastatin (5 mg/Kg/day, dissolved in 0.1% methanol in the drinking water), starting 48 h before AngII-infusion. Control groups of animals without treatments were also evaluated. We have studied 10 animals per group. Blood pressure was measured by tail-cuff pletysmography. Neither atorvastatin nor simvastatin modify blood pressure in AngII-infused rats (not shown). All experimental procedures were approved by the Animal Care and Use Committee of the Fundación Jimenez Diaz Institute, according to the guidelines for ethical care of experimental animals of the European Community (RD 223/88 MAPA and 609/86).

### Cell cultures

VSMCs were obtained from thoracic aorta of Wistar rats by collagenase method [15]. VSMC from passages 2 to 7 were used showing >99% positive immunostaining against smooth muscle α-actin (not shown). For experiments, cells at 80% confluence were arrested by serum-starvation for 48 hours. Cells were pretreated with Simvastatin (Sigma) or atorvastatin (kindly donated by Pfizer Madrid, Spain) for 48 hours and then treated with 10^−7^ mol/L AngII (Fluka, Sigma), for 20 minutes or 24 hours. For kinase experiments, cells were incubated for 1 hour with the following MAPK inhibitors: SB203580 (p-38 inhibitor; 10^−6^ mol/L), U0126 ethanolate (MEK1/2 inhibitor: 10^−5^ mol/L) (Promega), and SP600125 (JNK inhibitor; 10^−5^ mol/L), from Stressgen Bioreagents Corp; or with the ROCK inhibitor Y-27632 and Fasudil (10^−6^ mol/L; (TOCRIS), and then treated with 10^−7^ mol/L AngII, or 1 ng/ml human recombinant TGF-β1 (Peprotech) for 20 minutes. None of the inhibitors or statins were toxic at the doses used (evaluated by cell viability assay MTS-PMS, Promega), neither modify Smad proteins levels or activation.

### Protein studies

To quantify protein levels Western blot was done [12]. Equal protein loading was determined by BCA method (Pierce). The autoradiographs were scanned using the GS-800 Calibrated Densitometer (Quantity One, Bio-Rad), and data was calculated as n-fold over control. TGF-β1 protein levels were measured using a commercial enzyme-linked immunoassay (ELISA) (Bioscience) following the manufacturer's instructions. TGF-β1 activity was quantified by comparison with a standard curve using increasing concentrations of human TGF-β1.

For *in vivo* studies, paraffin-embedded sections of rat aorta were studied by immunohistochemistry. Briefly, after Antigen Retrieval and blockade of endogenous peroxidase aorta sections were incubated with primary antibodies overnight at 4°C. After washing, slides were treated with the corresponding anti-IgG biotinylated-conjugated secondary antibody (Amersham Bioscience) followed by the avidin-biotin-peroxidase complex (Dako), and 3,3′-diaminobenzidine as chromogen. Sections were counterstained with Carazzi's hematoxylin. The specificity was checked by omission of primary antibodies and use of non-immune sera. Immunohistochemistry was analized by Imagepro-plus, Media Cybernetics; Inc. All samples were evaluated in a blinded fashion. For each rat, the mean score value was obtained by evaluating 4 different high-power fields (x 20) per section.

For *in vitro* studies, immunocytochemistry was done in cells growing in coverslips, fixed with Merckofix (Merck), treated with 0.1% Triton-X100 and incubated with primary antibodies followed by ALEXA 488 labeled antibody. Nuclei were stained with DAPI. The absence of primary antibody was the negative control. Samples were mounted in Mowiol 40-88 and examined by a laser scanning confocal microscope (Leika). The experiments were done with 3 different cell culture preparations.

Antibodies employed were: pSmad2 (Cell signaling), Smad4 (Santa Cruz Biotechnology), tubulin (Sigma-Aldrich), fibronectin, type I collagen and G3PDH (Millipore).

Southwestern histochemistry was done using the consensus Smad sense (5′-GAGTATGTCTAGACTGACAATGTAC -3′) probe and controls previously described [7].

### Gene studies

Total RNA was isolated with Trizol method (Gibco) and gene expression was analyzed by Real-time PCR, performed on ABI Prism 7500 sequence detection PCR system (Applied Biosystems) according to manufacturer's protocol. Assay IDs used were TGF-β: Rn00572010_m1, TIEG: Rn00579697_m1; CTGF: Rn00573960_m1, plasminogen activator inhibitor-1 (PAI-1): Rn00561717_m1; type I collagen: Rn00584426_m1; metalloproteinase-9 (MMP-9), Rn00579162_m1; tissue inhibitor of matrix metalloproteinase-1 (TIMP-1), Rn00587558_m1; fibronectin: Rn00569575_m1 (Applied Biosystems, Foster City, CA). Data were normalized with 18S eukaryotic ribosomal RNA: 4210893E. Each animal was evaluated independently and data were expressed as mean±SEM as n-fold increase vs. control group.

### Statistical analysis

Results throughout the text are expressed as mean±SEM. One-way analysis of variance was used to determine differences between agonist-treated groups and controls. When statistical significance (p<0.05) was found, post-hoc Bonferroni or Dunnett tests were used to identify group differences. Statistical analysis was conducted using the SPSS statistical software, version 11.0 (SPSS).

## Results

### AngII directly, by a TGF-β independent mechanism, activates Smad pathway and increases ECM-related proteins in rat aorta

**Figure 1:**
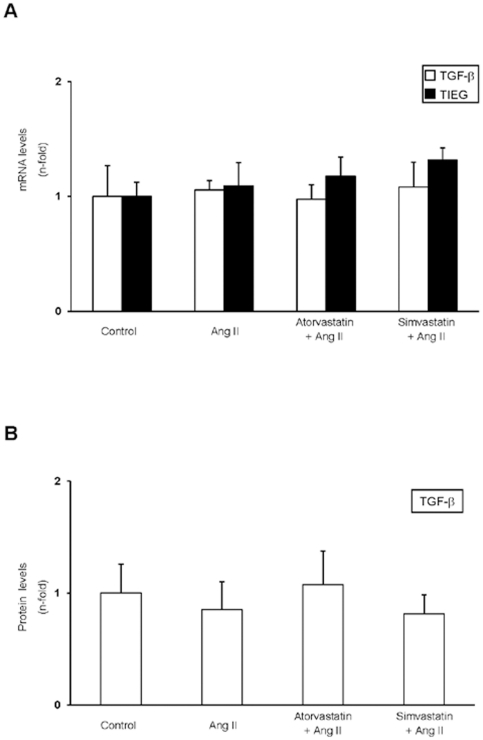
TGF-β is not upregulated in the aorta of AngII-infused rats. Rats were infused with AngII (100 ng/kg per minute, subcutaneously) for 3 days. Some animals were also treated with simvastatin or atorvastatin (5 mg/Kg/day), starting 48 hours before AngII infusion. **A.** RNA was isolated from frozen samples of rat aorta and gene expression was evaluated by real time and expressed as mean±SEM of 10 animals per group. **B.** TGF-β protein levels were measured in aortic protein extracts by Elisa. Data are expressed as mean±SEM of 10 animals per group.

**Figure 2:**
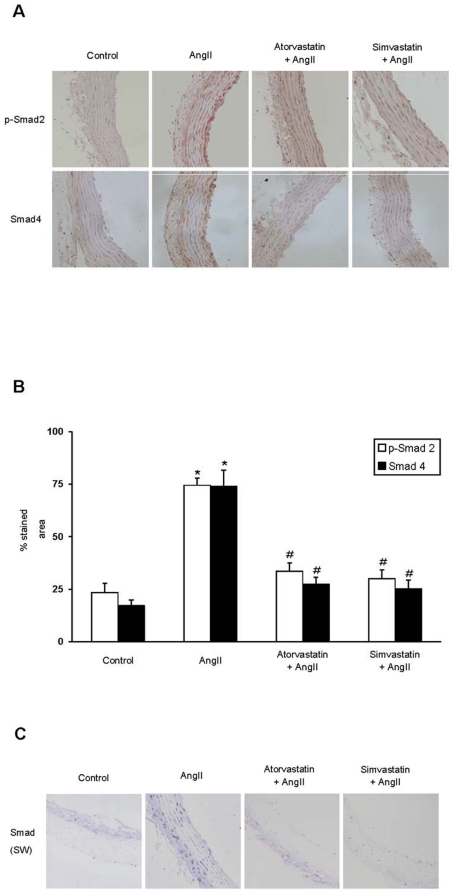
AngII activates the Smad pathway in rat aorta: Inhibitory effect of statins. Aortic sections were studied by immunohistochemistry using antibodies against phosphorylated Smad2 (p-Smad2) and Smad4. Figure A shows a representative immunohistochemistry experiment and B the quantification expressed as mean±SEM of 10 animals studied per group, *p<0.05 vs control; #p<0.05 vs AngII. C. Southwestern histochemistry evaluated Smad-dependent DNA binding activity. The arrow shows active nuclear Smad complexes that were mainly found in AngII-infused samples (Magnification 200×).

Previous studies have shown that AngII activates Smad signaling pathway in rat aorta [5], [6], but whether AngII-induced Smad activation was a direct effect or mediated by endogenous TGF-β production was not elucidated. After 3 days of AngII infusion we have observed that there was no increase on aortic mRNA or protein levels of TGF-β1 (Figure 1A and B), while Smad pathway is activated (Figure 2), as described [5]. We also evaluated the expression of TGF-β-inducible early gene-1 (TIEG), one of the earliest events in TGF-β mediated Smad signalling [16]. In AngII-infused rats TIEG gene levels were not modified (Figure 1A). These data indicate that Smad activation occurs earlier than TGF-β upregulation, suggesting that *in vivo* AngII directly activates the Smad pathway in the aorta.

**Figure 3:**
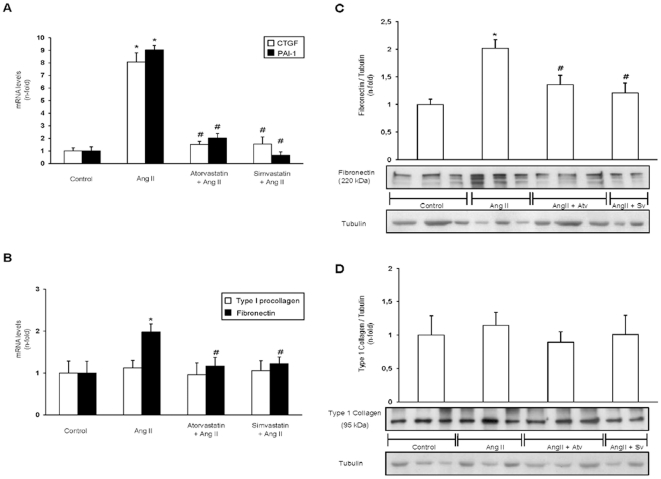
Infusion of AngII for 3 days increases ECM-related proteins in rat aorta: Inhibitory effect of statins. Gene expression of profibrotic factors CTGF and PAI-1 (A) or ECM proteins, fibronectin and type I procollagen (B) was evaluated by real time PCR. Figure C and D show a representative Western blot and data of aortic fibronectin and type 1 collagen protein levels, respectively. Data are expressed as mean±SEM of 10 animals per group, *p<0.05 vs control; # p<0.05 vs AngII.

**Figure 4:**
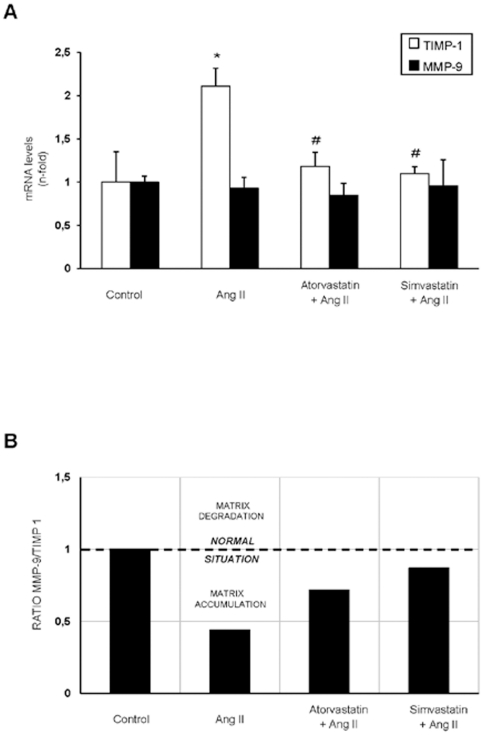
Regulation of matrix metalloproteinases and its inhibitors in the aorta of AngII-infused rats: Restoration by statins. **A**. Gene expression of MMP-9 and TIMP-1 was evaluated by real time PCR and data are expressed as mean±SEM of 10 animals per group, *p<0.05 vs control; # p<0.05 vs AngII. B. Evaluation of matrix balance by the ratio MMP-9 vs TIMP-1. Values higher or lower than 1 reflect degradation or accumulation, respectively, of matrix proteins.

Next, we have characterized the effect to AngII infusion for 3 days in the vascular wall evaluating ECM-related proteins. In frozen samples of rat aorta, upregulation of the profibrotic factors CTGF and PAI-1 was found at gene expression levels (Figure 3A). Regarding ECM proteins, overexpression at mRNA levels of fibronectin, but not type I procollagen, was observed (Figure 3B). Aortic fibronectin deposition was found at 3 days of AngII infusion (Figure 3C), while there was no change on type I collagen deposition, observed by western blot (Figure 3D), and by sirius red staining (not shown). The balance between synthesis and degradation regulates ECM accumulation; a process controlled by matrix metalloproteinases (MMPs) and their tissue inhibitors (TIMPs). Gene levels of MMP-9, a collagen-degrading enzyme, were not modified in response to AngII treatment, while aortic TIMP-1 expression levels, the main inhibitor of MMP-9 [17], were upregulated (Figure 4A). The ratio of MMP-9/TIMP-1 indirectly defines MMP-9 activity [18]. As seen in figure 4B, in AngII-infused rats the MMP-9/TIMP-1 ratio in aorta was around 0,4-fold showing a clear matrix accumulation environment. These results show that AngII *in vivo* activates Smad pathway and upregulates profibrotic factors and ECM proteins, by a TGF-β-independent process.

### Statins inhibits the activation of Smad pathway and the upregulation of ECM-related proteins observed in the aorta of AngII infused rats

To evaluate the effect of statins on AngII-induced Smad activation, rats were treated with simvastatin or atorvastatin (5 mg/Kg/day), starting 48 hours before AngII infusion and studied after 3 days. Both statins diminished Smad activation caused by AngII in the aorta, as observed by decreased levels of phosphorylated Smad2 and Smad4 by immunohistochemistry (Figure 2A and B). By Southwestern histochemistry we have confirmed the inhibitory effect of statins on AngII/Smad activation. In AngII-infused rats for 3 days, a nuclear blue staining, indicating active Smad complexes that bind to a consensus Smad sequence in DNA, was observed in many vascular cells, while few positive cells were found in control, simvastatin or atorvastatin treated samples (Figure 2C).

The overexpression of profibrotic factors and ECM components observed in AngII-infused rats at gene and protein levels were downregulated by treatment with simvastatin or atorvastatin (Figure 3). Moreover, the MMP-9/TIMP-1 ratio was reverted to levels observed in control rats (Figure 4). In contrast, there was no differences on TGF-β aortic levels between controls, AngII-infused or statins-treated groups (Figure 1). All these data shows that both statins diminished the direct (TGF-β-independent) activation of Smad pathway by AngII and subsequent upregulation of ECM-related factors in rat aorta.

### Statins inhibits the Smad pathway activation by AngII in cultured vascular smooth muscle cells

**Figure 5:**
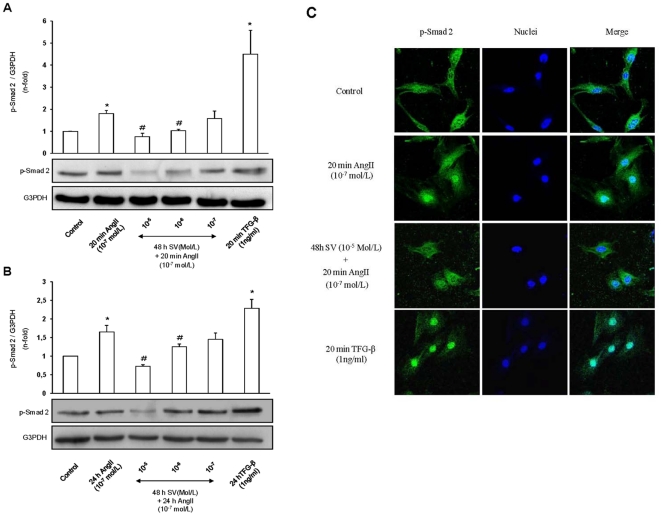
Statins inhibit the AngII/Smad pathway in cultured vascular smooth muscle cells. Cells were pretreated with simvastatin (SV) for 48 hours before stimulation with AngII for 20 minutes (A, C) or 24 hours (B). Figures A and B show a representative Western blot and data as mean±SEM of 3–6 independent experiments. *p<0.05 vs. control, #p<0.05 vs. AngII. Figure C shows a representative confocal microscopy experiment of 3 independent observations.

Preincubation of VSMCs with simvastatin significantly inhibited, in a dose-dependent manner, AngII-induced Smad2 phosphorylation observed at 20 minutes and 24 hours (Figure 5A and B, western blot). By confocal microscopy, we have observed that simvastatin also inhibited the nuclear localization of p-Smad2 caused by incubation with AngII for 20 minutes. As can be seen in the overlaid images of Figure 5C, AngII-treated cells presented a white tone in the nucleus indicating p-Smad2 nuclear localization. This nuclear localization was inhibited by simvastatin. Similar inhibitory effects were observed with atorvastatin (data not shown). These results show that in cultured VSMCs statins also inhibited AngII-induced Smad activation, at all times studied.

### Molecular mechanisms involved on statins inhibition of the AngII/Smad pathway

**Figure 6:**
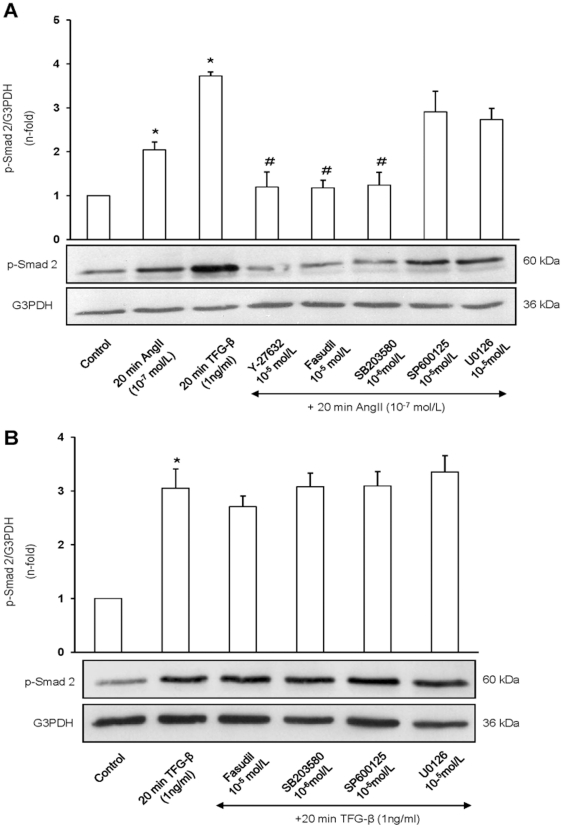
p38 MAPK and ROCK inhibition disminishes AngII mediated Smad2 phosphorylation in VSMCs. Cells were preincubated for 1 hour with the inhibitors before treatment with AngII (A) or TGF-β (B) for 20 minutes. Figure shows a representative Western blot and data as mean±SEM of 5–6 independent experiments. *p<0.05 vs control; # p<0.05 vs AngII.

We have previously described that statins inhibit several AngII-induced intracellular responses, including MAPK and RhoA/ROCK activation [14]. To test whether statins inhibitory effect on Smad was due to the modulation of these kinases a pharmacological approach in VSMCs was done. Only the p38 MAPK inhibitor SB203580, but not extracellular signal-regulated kinase1/2 (MEK/ERK) (U0126) or Jun N-terminal kinase (JNK) (SP600125), diminished AngII-induced Smad2 phosphorylation (Figure 6A). To assess the implication of the small G protein RhoA in the AngII/Smad pathway activation, VSMCs were preincubated for 1 hour with two selective inhibitors of serine/threonine ROCK I and II (Y-27632 and Fasudil). ROCK inhibition decreased the phosphorylation of Smad2 caused by AngII (Figure 6A). These data clearly show that p38-MAPK and RhoA/ROCK participates in AngII/Smad activation. The above-described inhibitory effects could explain the molecular mechanism involved in statins effect on the regulation of AngII/Smad pathway in vascular cells.

We have recently demonstrated that statins enhace Smad activation caused by TGF-β through the inhibition of RhoA and its downstream kinase ROCK [12]. For this reason, we have evaluated whether activation of RhoA/ROCK or MAPK cascade could be involved in TGF-β/Smad activation in VSMCs. None ROCK or MAPKs inhibitors modified the increased p-Smad2 in response to TGF-β (Figure 6B), suggesting that TGF-β/Smad activation is independent of these kinases.

## Discussion

The data presented here demonstrate that AngII induces Smad activation in the rat aorta by a direct, TGFβ independent, process, and regulates several Smad-dependent proteins involved in vascular fibrosis. This study extends the previous *in vitro* data to an *in vivo* model and remark the importance of AngII as a direct activator of Smad and inducer of profibrotic factors involved in pathological accumulation of EMC components in the vasculature.

We have previously shown that infusion of AngII into rats for 3 days activates Smad pathway in the aorta [5], as we have confirmed here. Previous studies have described that AngII increases TGF-β synthesis in rat aorta after 7 days associated to elevated Smad2 phosphorylation [10]. In the present study, we have found that at 3 days of AngII infusion aortic TGF-β, both at gene and protein levels, was not upregulated while Smad signaling pathway was activated. Previous studies have shown that in cultured VSMCs AngII caused a direct and rapid activation of Smad pathway [5], [6]. All these results show that AngII directly, by a TGF-β-independent process, activates the Smad pathway both *in vivo* and *in vitro* in the vasculature. *In vitro* studies have shown that Smad pathway is involved in AngII-induced fibrosis. Smad7 overexpression inhibited AngII-induced CTGF, fibronectin and collagen expression in VSMCs [5], and Smad2-siRNA prevented type I collagen network formation induced by AngII [19]. CTGF is an important mediator of ECM accumulation by AngII in the vasculature [15]. The regulation of CTGF by AngII has been extensively studied, involving activation of Smad, protein kinases (MAPKs and ROCK) and redox process [15], [20]. In cultured VSMCs, TGF-β also induces CTGF production [4]. In rats infused with AngII for 3 days, we have observed aortic CTGF, but not TGF-β, upregulation at the same time of Smad activation. Pharmacological inhibition of PAI-1 protects against AngII-induced aortic remodeling [21]. We have found that PAI-1 is another important profibrotic factor induced by AngII via Smad, in a TGF-β indepedent manner. All these data suggest that CTGF and PAI-1 are early profibrotic mediators of AngII-mediated vascular fibrosis directly regulated by Smad pathway, by a TGF-β independent process.

Several groups have characterized the histological changes caused by systemic infusion of AngII into rodents at the vascular wall. Only after 7 days of AngII infusion accumulation within the arterial wall of ECM proteins, including collagens, fibronectin and laminin, has been described [10], [15]. AngII infusion for 6 days increased aortic TIMP-1 expression in the absence of collagen deposition [22]. The synthesis of collagen is a complex process, regulated at gene and protein levels, including postraslational modifications and cross-links, being the net collagen levels also controlled by MMPs activity. We have observed elevated TIMP-1 gene levels, while MMP-9 mRNA levels were not modified, showing that after 3 days of AngII infusion the balance between ECM synthesis and degradation is shifted to matrix accumulation. Fibronectin is another key component of the extracellular matrix, overexpressed in hypertension-induced vascular damage [15], [23]. We have observed increased aortic fibronectin levels at 3 days of AngII infusion. In VSMCs, fibronectin release is increased by AngII and regulated by TGF-β, CTGF [15] and Smad [5]. Our data suggest that Smad and CTGF could be involved in the *in vivo* upregulation of fibronectin caused by AngII. In summary, several profibrotic factors and ECM related proteins are regulated by AngII/Smad pathway, independently of endogenous TGF-β synthesis, showing that direct activation of Smad by AngII is involved in the onset of vascular fibrosis.

Another important point of the present study is the demonstration the statins inhibited AngII-induced direct Smad activation in rat aorta. In cultured VSMCs statins also blocked the direct AngII/Smad activation, observed at 20 min, supporting our *in vivo* findings. Statins treatment downregulates profibrotic factors (CTGF, PAI-1) and ECM-related proteins (fibronectin) induced by AngII in rat aorta, and restores ECM synthesis/degradation balance to a normal situation. These data suggest that blockade of AngII/Smad pathway could be a mechanism involved in the beneficial effects of statins on vascular fibrosis.

We have further investigated the molecular mechanisms involved in the direct activation of AngII/Smad pathway, using a pharmacological approach in cultured VSMCs. We have observed that inhibition of p38 MAPK and ROCK participates in the activation of AngII/Smad pathway. Statins, acting at cellular level, inhibited several intracellular pathways. In particular, in VSMCs statins inhibited AngII-induced MAPK and RhoA/ROCK activation [14], [24]. All these data suggest that the mechanisms involved in the inhibitory effect of statins on AngII/Smad, could be due to the modulation of RhoA/ROCK and MAPK activation.

Previous studies have demonstrated that statins down-regulate vascular TGF-β over-production caused by AngII, both *in vitro* and *in vivo* [14]. In VSMCs, AngII induced a long-term Smad activation mediated by endogenous TGF-β synthesis [6]. In these cells we have observed that simvastatin inhibited AngII-induced Smad activation observed at 24 hours. In a model of chronic AngII infusion for 2 weeks, we have previously shown that atorvastatin inhibited vascular fibrosis and aortic TGF-β upregulation [14], associated to Smad pathway inhibition (unpublished observations). All these data suggest that statins inhibited AngII-induced endogenous TGF-β synthesis and subsequent Smad activation observed in long time studies. Our data is supported by findings in a mice model of AngII-induced cardiac damage. Treatment with another statin, pitavastatin, inhibited TGF-β production and Smad2/3 phosphorylation in cardiac tissue and exerts protective effects on cardiovascular remodeling, diminishing myocardial interstitial fibrosis [25].

In contrast, the effect of statins on TGF-β/Smad regulation in atherosclerosis is different. In the ApoE knockout mice model of atherosclerosis we have recently found that atorvastatin increased Smad3 phosphorylation and ECM-related proteins, including CTGF, PAI-1 and type I collagen in the fibrous cap [12]. In ApoE/LDLR double knockout mice atorvastatin also increased phosphorylation of Smad2/3 in aortic endothelium covering atherosclerosis lesions [13]. In cultured VSMCs statins enhance Smad pathway activation by TGF-β leading to an increase in TGF-β-dependent actions, including accumulation of ECM proteins [12]. These data suggest that the protective effect of statins in plaque stability could be due to the enhancement of TGF-β/Smad pathway. We have recently observed that RhoA/ROCK inhibition (by pretreatment for 48 hours with toxin C3 or ROCK inhibitors) increases Smad activation caused by TGF-β, by a process mediated by the upregulation of TGF-β type II receptor [12]. However, ROCK inhibition diminished AngII-induced Smad2 phosphorylation. These data show that RhoA/ROCK differentially regulates Smad activation elicited by AngII or TGF-β, and its inhibition mimics statins effects.

Smad pathway is the main TGF-β signaling pathway [16], [26]. TGF-β, through Smad2/4 signaling, contributes to vascular remodeling by VSMC growth and ECM synthesis at sites of vascular injury [27]. TGF-β/Smad2 regulates CTGF, as shown by Smad7 overexpression and studies with CTGF promoter [5], [28]. Although TGF-β/Smad is an important pathway in vascular fibrosis, other factors and signaling systems are also involved. In VSMCs, TGF-β regulates PAI-1 expression by the EGFR/pp60(c-src)/MEK-ERK pathway and independent of Smad2 activation [29]. We have observed that TGF-β-induced Smad2 phosphorylation was not modified by MAPKs inhibitors, confirming an ERK-independent Smad2 pathway in VSMCs. All these results indicates that statins differentially regulates Smad pathway activation by AngII or TGF-β in the vasculature in different pathological conditions.

TGF-β is implicated in many human fibrotic disorders. TGF-β is one of the most potent inducers of ECM proteins in fibroblasts and contributes to epithelial mesenchymal transition (EMT) in different tissues [30], [31]. Statins attenuates EMT in human tenon fibroblasts and renal tubuloepithelial cells, by inhibition of MAPKs activation, whereas Smad2/3 phosphorylation was preserved [32]–[34]. In contrast, simvastatin abrogates activation of intestinal fibroblasts by TGF-β through the inhibition of Smad activation [35]. These data clearly show that the effect of statins on Smad regulation depends on cell type and pathological condition.

Our *in vivo* data demonstrate that AngII directly activates Smad pathway in the vessel wall and regulates several Smad-dependent proteins involved in vascular fibrosis, by a direct, TGFβ independent process. Statins are known as one of the best option for the treatment of cardiovascular diseases. The present study demonstrates that two statins, atorvastatin and simvastatin, inhibited AngII-mediated Smad activation, both *in vivo,* in rat aorta, and in cultured VSMCs, showing an intracellular mechanism of statins action that regulates vascular fibrosis. Our data afford additional information to the well-established pleiotropic effects of statins and could explain part of the early beneficial effects of statins observed in patients without high cholesterol levels but with other risk factors [36].
